# Multiscale Hyperbolic Embedding for Cell Hierarchies in Large-Scale Bioinformatics Data

**DOI:** 10.1101/2025.09.29.679407

**Published:** 2025-10-01

**Authors:** Mingchen Yao, Anoop Praturu, Tatyana Sharpee

**Affiliations:** 1 Computational Neurobiology Laboratory, Salk Institute for Biological Studies, La Jolla, CA 92037, USA; 2 Department of Physics, University of California, San Diego, La Jolla, CA 92093, USA

## Abstract

The increasing size of datasets poses challenges for their visualization and interpretation, highlighting the need for scalable and effective analysis methods. Hyperbolic embedding have shown strong potential in capturing complex hierarchical structures across diverse systems. However, existing hyperbolic embedding methods typically operate with fixed curvature and have difficulties scaling to large datasets. To address these limitations, we propose MuH-MDS, a novel multiscale algorithm for hyperbolic multidimensional scaling that uses “adiabatic” approximation from physics to optimize local positions while keeping cluster centroid fixed. MuH-MDS improves computing time by 10^3^ compared to previous methods and is able to handle large datasets comprising over 80, 000 samples. We validate the method on a number of datasets, including a large-scale *C. elegans* embryogenesis scRNA-seq dataset with over 80,000 samples. Here, MuH-MDS uncovers intrinsic hierarchical structure, and achieves improved pseudotime inference and lineage analysis compared to UMAP and other methods. Unlike UMAP and t-SNE, which emphasize local structure at the expense of global coherence and metric accuracy, MuH-MDS preserves global hierarchy in a metrically faithful manner, maintaining key relationships across scales.

## Introduction

Many modern biological datasets, such as gene regulatory networks or single-cell transcriptomics, exhibit inherent hierarchical structures^1^. These hierarchies reflect underlying biological processes, like developmental lineages or molecular cascades with some genes or molecules exterting a predominant influence. Traditional Euclidean embedding, which evaluate pairwise distances uniformly across the dataset, often fail to capture such asymmetries and nested relationships^2,3^. In particular, they struggle to faithfully represent the few principal axes along which selective amplification or differentiation occurs. Hyperbolic space offers a compelling alternative. With its exponentially expanding number of states, it can be viewed as a continuous analogue of a tree structure, making it particularly well-suited for datasets with hierarchical organization^4,5^. In such embedding, hierarchical depth is naturally encoded along the hyperbolic radius. For example, the hyperbolic embedding of *C. elegans* embryogenesis sequencing data reveals the radial axis as an effective proxy for developmental pseudotime^3^. Compared to Euclidean approaches, hyperbolic embeddings incur less distortion when modeling global structures^6^, enhancing both interpretability and visualization of complex biological hierarchies^5^.

Previous methods have demonstrated the significant advantages of hyperbolic embeddings in uncovering complex hierarchical structures in both graphs^7,8,9,10,11,12,13,14^ and biological data^5,3,6^. However, these methods are often constrained to fixed dimensions and curvature or do not scale effectively to handle large datasets.

Advancements in sequencing technologies have led to datasets that are increasingly large-scale, high-dimensional, noisy, sparse, and characterized by correlated features. These properties pose significant challenges while simultaneously highlighting the unique advantages of hyperbolic embedding algorithms. The large number of samples and sparse nature of scRNA-seq data complicate analysis and dimensional reduction, yet they have driven the development of sophisticated algorithms^15,16,17,3,5,18,19^ and provide abundant benchmark datasets for testing^20,21,22,23^. The inherently correlated expression of genes implies a low-dimensional intrinsic gene expression space, motivating these algorithms to extract valuable biological insights through dimension reduction, clustering, and pseudotime analysis. However, methods operating in Euclidean space, such as UMAP^24^ and t-sne^25^, often distort global data structures when projecting into low-dimensional space, potentially misplacing important samples or introducing artificial structures. The inherent complexity and hierarchical structure of biological processes render the resulting datasets intrinsically tree-like, making them difficult to represent without distortion in Euclidean space. In contrast, hyperbolic embedding methods are particularly well-suited for analyzing such data.

To address these challenges and leverage the advantages of hyperbolic space, we developed MuH-MDS, a multiscale hyperbolic multidimensional scaling embedding method. Building on the metric Bayesian hyperbolic multidimensional scaling (BHMDS) algorithm^12^, MuH-MDS introduces a novel global-local embedding framework to improve scalability. This method effectively approximates large distance matrices and significantly enhances the scalability of metric hyperbolic embedding, enabling its application to bioinformatics datasets and facilitating the discovery of meaningful hierarchical insights.

## Results

### Multiscale hyperbolic-MDS algorithm

**Figure 1:**
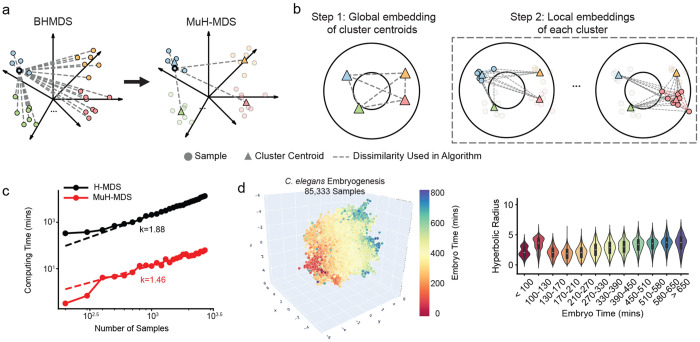
Multiscale hyperbolic multidimensional scaling (MuH-MDS) algorithm. (a-b) Schematic representation of (a) the distance approximation and (b) the global-local embedding. (c) Comparison of computing time between the original (BHMDS) and multiscale (MuH-MDS) approaches, using the mouse myeloid progenitors scRNA-seq dataset (“Paul”) with 2,730 samples^22^. (d) Left: Embedding of the full *C. elegans* dataset (85,333 samples) using the multi-layer approach in 3D hyperbolic space. MuH-MDS parameters: $N_{\mathrm{neighbors}}=30$, $N_{\mathrm{cluster}}=800$. Right: The embedding preserves the correlation between hyperbolic radius and embryo time in *C. elegans*.

The major challenge in creating scalable embedding methods for non-Euclidean spaces is that these methods can no longer rely on linear operations. Evaluating nonlinear distances across the whole dataset at each step of optimization becomes computationally prohibitive. Some methods use local linearization of the space^26^ to get access to linear algebra methods that have been optimized for speed. But this leads to global distortions. In contrast, we propose here to simplify most of the distance calculations by substituting distances between disparate samples with their distance to centers of other clusters (Fig. 1a). The key concept of the multiscale approach is to partition the samples into clusters before embedding and then perform the embedding in a “global-local” hierarchical sequence (Fig. 1b, Methods). In the global embedding step, one sets cluster centroid positions (Fig. 1b, left). Then, samples in each cluster are embedded locally, based on intra-cluster distancing and their distances to the nearest cluster centroids (Fig. 1b, right). Distance is estimated under the assumption that distances between clusters are much larger than distances within clusters, which is guaranteed by most clustering methods. This structure greatly reduces the embedding matrices’ size in each step and allows the local embedding step to be parallelized, significantly enhancing computational efficiency. We tested MuH-MDS and found that it provides a valid and efficient approximation (Methods), thereby making the algorithm scalable to much larger datasets.

### Scalability and reliability

We first compared the performance of MuH-MDS with the previous BHMDS algorithm, which has *O*(*n*^1.88^) computing time with the number of samples *n* to be embedded (Fig.1c). This result aligns with expectations, as the number of pairwise distances scales as *O*(*n*^2^). MuH-MDS significantly improves scalability, with computing time scaling as *O*(*n*^1.46^) (Fig. 1c). This is close to the theoretical optimal scaling factor of 1.33 (Methods). Meanwhile, by evaluating embedding quality using metrics such as *Q*_local_, *Q*_global_ (Methods), and the correlation between original pairwise distances and embedded distances, we demonstrated MuH-MDS retained high embedding quality (Fig. S1). While the computing time was reduced by a factor of 10^3^ (from 13,806 minutes for BHMDS to 15 minutes for MuH-MDS), the embedding quality metrics only dropped by less than 10% (Fig. S1c).

MuH-MDS enables the embedding of the full *C. elegans* dataset (85,333 samples) in 1.1 hours on a single CPU. For comparison, the Poincaré map requires 2-3 hours on 1 GPU for 40,000 samples^3^. MuH-MDS perfectly captures the correlation between embryo time and hyperbolic radius (Fig. 1d).

### Robustness to hyperparameter settings

**Figure 2:**
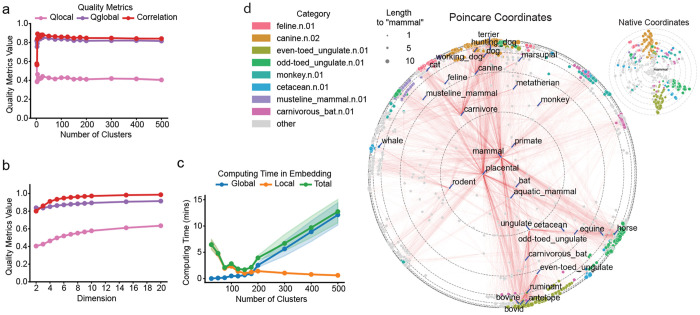
(a) Embedding quality metrics are robust across different numbers of clusters, except for small number of clusters. (b) Embedding quality metrics increase with the dimensionality of the hyperbolic space, with the number of clusters *k* = 100. (c) Trade-off between global and local computing time for simulations in (a). The embedding dimension is fixed at *D* = 3 for both (a) and (c). All simulations in (a)-(c) are conducted with 20 repetitions and number of neighbors *α* = 20. (d) Application of MuH-MDS to the WordNet mammal subtree. Marker size reflects the graph distance to the root node “mammal”, while color denotes membership in semantic categories as shown in the legend. These categories correspond to parent nodes with graph distance 3 to root node “mammal” and with the largest numbers of child nodes. They were selected to highlight major structural branches within the hierarchy. Red lines connect each node with all of its child nodes. Inset: Same embedding shown in native coordinates, which visually emphasizes child nodes. MuH-MDS parameters: *k* = 198, *α* = 60.

**Figure 3:**
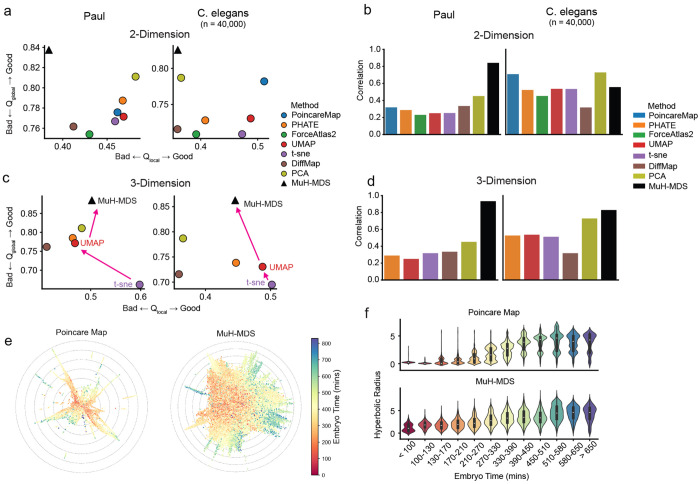
(a-d) Embedding quality metrics for two datasets: Paul^22^ and *C. elegans*^23^, compared with competing methods, in 2D (a-b) and 3D (c-d) spaces. (e) 2D embeddings of the *C. elegans* dataset (40,000-sample subset) using the Poincaré map and MuH-MDS. (f) MuH-MDS more uniformly captures the correlation between hyperbolic radius and embryo time. (See Fig. S4)

We then evaluated how hyperparameter settings affect the embedding quality metrics and computing time. Several parameters were tested: (1) the number of clusters in the clustering step (Fig. 2a,c), (2) the dimensionality of the hyperbolic space into which the data is embedded (Fig. 2b), (3) the maximum and minimum (Fig. S2a–b) allowed cluster size and (4) the number of nearest neighbors in the local embedding step (Fig. S2c). We found that embedding quality was only sensitive to the number of nearest neighbors for high embedding dimension (Fig. S2c), since more neighboring points are needed as constraints to determine locations in higher dimensionality. Most embeddings intended for visualization purposes are in 2D or 3D spaces, where sensitivity to the number of nearest neighbors can be safely neglected.

#### Number of clusters (*k*): computing time trade-off between global and local embedding.

Embedding quality metrics were sensitive to the number of clusters only when the number of clusters was very small (*k* < 10, Fig. 2a). Beyond this number, embedding quality remained stable, varying by approximately ±5%. This is because *k* ≈ 10 cluster centroids is usually sufficient to determine the global shape of the manifold when embedding into two or three dimensions. By comparison, computing time varied significantly with the number of clusters. There was an optimal number of clusters that minimized computing time. The optimal number of clusters scaled with the number of samples as *k* = *n*^2/3^ to balance the global and local embedding steps. With the optimal number of clusters, the algorithm achieved the best computing time *O*(*n*^1.33^) (Methods). This is consistent with Fig. 1c, where computing time scales as *O*(*n*^1.46^), and with Fig. 2c, where the best computing time is observed between *k* = 100 and *k* = 200, aligning with the theoretical *k* = 2730^2/3^ = 185. Choosing the optimal number of clusters wisely, which can be easily controlled in the clustering step for most clustering methods, significantly optimizes the computing time of MuH-MDS.

#### Dimension of the space: 3D is sufficient to characterize global features.

Embedding quality increases monotonically with the dimensionality of the hyperbolic space (Fig. 2b), as higher dimensions provide more capacity to capture details in the data. Notably, while the local embedding quality *Q*_local_ continues to improve with increasing dimensions, the global embedding quality *Q*_global_ and the Shepard diagram correlation saturate at *D* = 4. This indicates that hyperbolic space effectively captures most of the global information within the data in just 2 or 3 dimensions. These results highlight the advantage of hyperbolic embedding for visualizing complex, high-dimensional data in low-dimensional spaces.

#### Cluster size distribution control.

To ensure more efficient embedding, we control the size distribution of clusters during the embedding process. For very small clusters, for example, those with only 1-2 samples in a cluster, we apply an “exclude and remap” procedure to reduce the total number of clusters in the global embedding step. Specifically, small clusters are excluded from the global-local embedding process and are mapped to the embedding space only after all other points have been embedded (Methods). For very large clusters that could significantly prolong local embedding time, we further subdivided them into smaller clusters until each sub-cluster size was within the maximum allowed limit. This cluster size distribution control significantly reduced embedding time (Fig. S2b) and helped improve embedding quality (Fig. S2a). By excluding clusters that are too small, the algorithm focuses on capturing the geometry of the majority of points during the embedding step, leading to a more precise representation of the global geometry.

### Embedding of graph datasets

While most of our experiments focus on scRNA-seq data, we include several examples of non-biological graph datasets to demonstrate that MuH-MDS performs well across diverse types of data. The classical problem of word embedding serves as an illustrative example of complex network data, where distances are defined as paths in the graph. Using the mammal tree from the WordNet dataset^27^, we computed pairwise distances between samples in the graph and performed 2D hyperbolic embedding using MuH-MDS (Fig. 2d). The resulting distortion rate was 0.158, which is comparable to previous embedding methods^10^. Nodes with a smaller distance to the root “mammal” are placed at smaller radius, and vice versa. When represented in native coordinates (Fig. 2d, inset), it is clearer that MuH-MDS gives an embedding that represents node depth in the mammal closure as radial direction, and different categories as angular direction, giving a clear representation of the tree. Our embedding better preserves hierarchical data structures. For instance, feline and canine nodes are positioned close to carnivore, a relationship that was not captured by methods such as HoroPCA^10,11^. This advantage arises from the metric embedding approach, which maintains the relative scale of distances between samples. Additional results for graph dataset embeddings are shown in Fig. S3. Embedding graph data into a 2D space using MuH-MDS effectively represents the tree-like structure of the graph while preserving original graph relationships with minimal distortion.

### Performance evaluation on benchmark bioinformatics datasets

We evaluated MuH-MDS on multiple benchmark scRNA-seq datasets against popular dimensional reduction methods in both 2D and 3D spaces. Results are presented for two classic datasets: the Mouse myeloid progenitors dataset (“Paul”)^22^ and *C. elegans* embryogenesis^23^ (Fig. 3a–d). In addition, we tested four other datasets with varying hierarchical structures and dataset sizes (Methods; Fig. S4, S6–S20). In both 2D and 3D spaces, MuH-MDS achieves the best global embedding quality and demonstrates high correlation between original distances and embedded distances. In 3D space, MuH-MDS further improves embedding quality both locally and globally, outperforming competing methods. This is consistent with Fig. 2b, where MuH-MDS, as a metric embedding method, shows a substantial performance improvement when increasing the embedding dimensionality from 2D to 3D.

To demonstrate the ability of MuH-MDS to uncover hierarchical structures in large-scale data, we analyzed the *C. elegans* embryogenesis dataset, which contains 85,333 samples in total. To ensure scalability for comparison with Poincaré map^3^, we randomly sampled 40,000 cells for the analysis in Fig. 3. In this dataset, embryo time is recorded, serving as an ideal indicator of the hierarchy in the differentiation process, where smaller embryo ages correspond to the root of the tree and larger ages to the leaves. The Poincaré map demonstrates that the hyperbolic radius in the embedding correlates with embryo time, reflecting the tree-like structure inherent in the data. MuH-MDS provides a more continuous and well-distributed representation than the Poincaré map (Fig. 3e–f), with lower global distortion (Fig. 3a).

### MuH-MDS reveals hierarchy of cell lineages in *C. elegans* datasets

In bioinformatics, scRNA-seq data offers abundant information about sub-clusters and lineages. However, global structure is often distorted during dimensional reduction with methods such as UMAP when applied to the full dataset^5^. MuH-MDS leverages the properties of hyperbolic space, with its exponential expansion, to effectively capture both global and local structures in the same embedding compared to competing methods.

**Figure 4:**
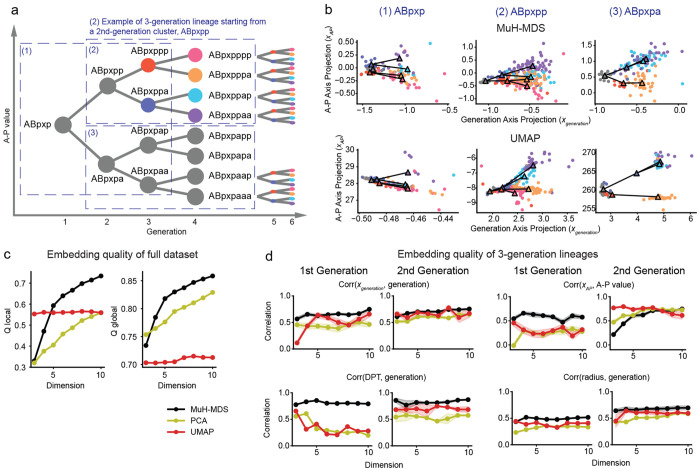
(a) Schematic representation of the ABpxp lineage, where “a” and “p” represent “anterior” and “posterior” in *C. elegans* development. Dashed boxes showed 3 examples of 3-generation lineage originating from parent nodes ABpxp, ABpxpp, and ABpxpa. (b) Visualization of the three example lineages in 2D space, projected from 7D embeddings of either MuH-MDS or UMAP (Methods). Triangles indicates cluster centroids of each population, and lines connecting the centroids. MuH-MDS preserves a better tree-like structure, while UMAP merges some clusters. MuH-MDS parameters: *α* = 20, *k* = 50. (c) Embedding quality metrics for the entire ABpxp lineage (7,562 samples) across different dimensions, compared between MuH-MDS, UMAP, and PCA. (d) Comparison of 4 sub-lineage embedding quality metrics: (1) correlation between generation and projected coordinates (Corr($x_{\text{generation}}$, generation)), (2) correlation between A-P value and projected coordinates (Corr(*x*_AP_, A-P value)), (3) correlation between generation and diffusion pseudotime inferred from embedding (Corr(DPT, generation)) and (4) correlation between generation and sample radius in embeddings (Corr(radius, generation)), across different dimensions, embedding methods (MuH-MDS, PCA, and UMAP), for the generation 1 lineage (ABpxp) and generation 2 lineages (ABpxpp and ABpxpa) (See Fig. S5 for more lineages). Across all embedding dimensions and quality metrics, MuH-MDS has a better performance compared with other methods, except for metric (2) for generation 2 lineages, where it obtains the best embedding quality after dimension 8 and keeps improving.

We tested MuH-MDS on a neuronal progenitor subset, the ABpxp lineage (7,562 samples) from the *C. elegans* dataset. Across varying embedding dimensions, MuH-MDS exhibited higher global embedding quality with dimension 4 and achieved higher local embedding quality starting with dimension 5 (Fig. 4c).

To evaluate the preservation of lineage relationships, both locally and globally, we examined 3-generation lineages originating from a single parent node. These parent nodes included global nodes (e.g., ABpxp) and local nodes (e.g., ABpxpppp). Samples within each 3-generation lineage were color-coded based on their relationship to the parent node (Fig. 4a). The representation was visualized in 2D space, mimicking typical visualization procedures, with the embedding projected onto a 1D subspace that best captured either generation depth or differentiation along the “anterior-posterior” axis (“A-P value”) (Fig. 4a, Methods). Samples were randomly split 20 times into 50% training and 50% test sets. For each split, the training set was used to determine the projection direction that best correlated with each metric, and the projection of the test set onto this direction was used to compute the correlation for evaluation. Intuitively, a representation exhibiting high correlations along both projection axes would closely resemble the underlying hypothetical tree structure shown in Fig. 4a, which is exactly what we see in MuH-MDS (Fig. 4b). Meanwhile, UMAP demonstrates strong clustering capabilities but tends to merge distinct and biologically important nodes, such as the ABpxppp and ABpxppa lineages (Fig.4b, middle column).

We computed the correlation between projections onto the generation axis (*x*_generation_) and actual generation depth, and the correlation projections onto the “anterior-posterior” axis (*x*_A-P_) and actual “A-P” value, across different embedding dimensions, for the ABpxp, ABpxpp, and ABpxpa lineages (Fig. 4d; See Fig. S5d for more lineages). MuH-MDS best preserved both generation and “A-P” value across all dimensions for 3-generation lineage from ABpxp, which is the most global lineage (Fig. 4d). It also performed best for ABpxpp and ABpxpa lineages for the generation representation and “A-P” value representation beyond dimension 6. While UMAP performed well for local parent nodes (Fig. S5d, last column), it failed to accurately represent global nodes (Fig. 4d). While methods like UMAP excel in local embeddings, they often distort global structure when applied to the entire dataset. Additionally, UMAP’s non-metric approach results in representations where generation and anterior-posterior relations may not align in biologically meaningful ways. In contrast, MuH-MDS efficiently preserved both global and local hierarchical structures. To better align with standard bioinformatics analyses, we computed pseudotime from each embedding, computed using diffusion pseudotime (DPT) based on the diffusion map constructed from the local neighborhood graph (Methods). DPT derived from MuH-MDS showed the strongest correlation with the ground truth generation depth across all dimensions and lineages. Since the hyperbolic radius can also serve as an indicator of developmental pseudotime, we further computed the correlation between sample radius and generation depth in each embedding. MuH-MDS again exhibited the highest correlation. For more localized lineages, the hyperbolic radius could be a better indicator of developmental progression than DPT (Fig.S5d). This may be because DPT infers temporal order by modeling diffusion processes over a k-nearest neighbor graph constructed from the data. The structure of this graph is sensitive to the choice of the number of neighbors: too few may lead to disconnected components, while too many may obscure local topology and distort temporal inference. In contrast, the radius offers a more stable geometric measure, as it does not rely on a discrete graph and instead captures continuous distance from the origin in hyperbolic space. In summary, MuH-MDS is a powerful method for preserving both global and local structures in datasets with complex hierarchical relationships.

## Discussion

Leveraging a multiscale global-local embedding approach, we developed MuH-MDS, a hyperbolic MDS embedding method that achieves both efficiency and scalability on large datasets. MuH-MDS uses adiabatic approximation to find local embedding positions within a cluster while keeping inter-cluster positions fixed. The metric property of the embedding ensures that the low-dimensional representation is quantitatively meaningful, while the well-defined hyperbolic geometry makes the method suitable for further analyses, such as identifying feature-correlated axes, subtypes, and lineages, as well as performing computations between vector representations of samples.

MuH-MDS is both reliable and efficient, and the concept of global-local approximation can potentially be generalized to other cases where large dissimilarity matrices require approximation under a nonlinear metric, a scenario that often poses scalability challenges.

Unlike traditional methods like PCA, which provide a well-defined vector space but often distort relationships between samples due to limited dimensionality, hyperbolic embedding addresses this limitation. By leveraging its exponentially expanding space along the radius, hyperbolic embedding effectively characterizes complex hierarchical data within constrained dimensional spaces.

We observed a trade-off between global and local embedding times, driven by the balance between cluster numbers and sizes. The main impact on computing time that can be achieved without compromising embedding quality is to select appropriate clustering parameters, with the number of clusters scaling as *n*^2/3^. Implementing additional cluster size distribution controls, such as re-dividing overly large clusters, has been shown to further reduce computing time while maintaining good embedding quality (Fig. S2).

The primary ways for future improvements of the algorithm is to allow for multiple rounds of updates in the centroid position. For current datasets, a single round has been sufficient, cf. Fig. 2b, but as the datasets size increases further, it might become necessary to consider multiple rounds of global updates. Another point that deserves careful consideration is the definition and computation of the original distances. When using a metric embedding algorithm, it is crucial to consider whether the chosen metric appropriately captures the desired features and operates at a reasonable scale. In scenarios where defining a meaningful metric is difficult or only rank-based relationships between samples are of interest, non-metric methods may be more appropriate. We note that the adiabatic approximation employ here to improve metric multidimensional scaling can be also be used to improve non-metric multidimensional scaling approaches^28^.

## Methods

### Hyperbolic geometry

The literature on hyperbolic geometry in complex systems is vast, and we do not intend to cover it in detail here. We cover only the basics necessary for the model and refer the reader elsewhere for more details^4^. We follow^2^ who found that using Lorentzian coordinates for hyperbolic space is significantly more computationally stable. In this model, a *D*-dimensional hyperbolic space is represented by a future facing space like sheet in a *D* + 1-dimensional Minkowski space-time, i.e., the set of points satisfying

(1) $$x_{0}^{2}-x_{1}^{2}-x_{2}^{2}\ldots -x_{D}^{2}=1$$

introducing the metric tensor $g_{\alpha \beta }=Diag(1,-1,-1,\ldots ,-1)$, where Greek indices run from 0 to *D*, and employing the Einstein summation convention where repeated indices are summed over, we can compactly express the constraint equation as $g_{\alpha \beta }x^{\alpha }x^{\beta }=1$. Continuing with this notation, the distance between two points $x^{\alpha }$ and $y^{\beta }$, which satisfy the constraint equation, is

(2) $$d_{xy}=\text{arccosh}\left(g_{\alpha \beta }x^{\alpha }y^{\beta }\right)$$

In practice, the *D* space-like components $\vec{x}$ of the coordinates were taken as free parameters, and the time-like component *x*_0_ was computed according to the constraint equation $x_{0}=\sqrt{1+\vec{x}\cdot \vec{x}}$.

While coordinates in the model are with unit curvature *K* = −1, the curvature can be effectively fit by fitting for an overall scaling factor of the embedded distance matrix. This is because in curved spaces, the strength of the effect of curvature depends on the scale at which you observe the data. We refer the reader to^12^ for the mathematical details of this approach.

### Bayesian hyperbolic multidimensional scaling algorithm

MuH-MDS is based on a Bayesian Hyperbolic MDS algorithm (BHMDS)^12^. Briefly, the generative model assumes the data $\delta_{ij}$ are generated from a geometric distance matrix $d_{ij}$ in hyperbolic space by a noisy process, and subject to rescaling:

(3) $$\delta_{ij}=\frac{d_{ij}}{\lambda }+\epsilon_{ij}$$

Where $\epsilon_{ij}∼𝒩\left(0,\sigma_{ij}\right)$ are independent normally distributed variables with mean 0 and possibly differing variances. In practice, $\delta_{ij}$ was normalized so that $\text{max}\left(\delta_{ij}\right)=2$, so λ corresponds to the radius of the distribution of points in hyperbolic space and probes the effects of curvature (See^12^ for more details on this). From this generative model, the likelihood is

(4) $$ℒ\left(\left\{{\vec{r}}_{n}\right\},\lambda ,\left\{\sigma_{ij}\right\}\right)=\underset{i<j}{\prod }P\left(\delta_{ij}|{\vec{r}}_{i},{\vec{r}}_{j}\right)=\underset{i<j}{\prod }\frac{1}{\sqrt{2\pi \sigma_{ij}^{2}}}{\text{e}}^{\frac{1}{2\sigma_{ij}^{2}}{\left(d_{ij}/\lambda -\delta_{ij}\right)}^{2}}$$

As discussed by BHMDS^12^, the number of uncertainty parameters was reduced by assuming each point has a characteristic uncertainty $\sigma_{i}$ with an inverse gamma prior, and compute $\sigma_{ij}^{2}=\sigma_{i}^{2}+\sigma_{j}^{2}$. This has an interpretation in terms of springs connected in series^12^. We assume a flat prior for the embedding coordinates and put a normal prior on λ. Since the log-likelihood scales as N(N−1)/2, the log prior on λ was multiplied by N(N−1)/2 so that the strength of the prior is not reduced in the large *N* limit. Combining all of this, the negative log-posterior, i.e., loss function, is

(5) $$L=\frac{1}{2}\underset{i<j}{\sum }\left(\frac{{\left(d_{ij}/\lambda -\delta_{ij}\right)}^{2}}{\sigma_{i}^{2}+\sigma_{j}^{2}}+\text{ln}\left(\sigma_{i}^{2}+\sigma_{j}^{2}\right)\right)+\frac{N(N\;-\;1)}{4\sigma_{\lambda }^{2}}\lambda^{2}+\underset{i}{\sum }\left((a+1)\text{ln}\;\sigma_{i}+\frac{b}{\sigma_{i}}\right)$$

Where in practice^12^ we take $\sigma_{\lambda }=10$, *a* = 2, and *b* = 0.5. All parameters were fitted using an LBFGS optimizer implemented in the Stan statistical programming package.

### Global-local embedding procedure in the MuH-MDS algorithm

#### Clustering.

Before embedding, samples are divided into different clusters using classical clustering methods, such as k-means clustering. Denote a sample in *L*-dimensional feature space as ${\vec{v}}_{i}^{k}\in R^{L}$, where *i* denotes the sample index and *k* denotes the cluster it is assigned to. For cluster *k*, the cluster centroid was computed as $\vec{c_{k}}=\frac{1}{\left|A_{k}\right|}\sum_{\vec{v_{i}^{k}}\in A_{k}}\vec{v_{i}^{k}}$, where $A_{k}$ is the set of samples in cluster *k*.

#### Compute distance matrices.

The “global” embedding refers to the embedding of cluster centroids using traditional BHMDS, for which we need the distance matrix between cluster centroids $\delta_{mn}=|\vec{c_{m}}-\vec{c_{n}}|$. By definition, $\delta_{mn}$ is a *K*-by-*K* symmetric matrix, where *K* is the number of clusters. The “local” embedding fits each individual cluster $A_{k}$ using previously embedded centroid locations, for which we need two types of distances: (1) the distance between samples in cluster $A_{k}$, which is computed as $\delta_{k,ij}^{self}=|\vec{v_{i}^{k}}-\vec{v_{i}^{k}}|$, a $\left|A_{k}\right|$-by-$\left|A_{k}\right|$ symmetric matrix; (2) the distance from samples in cluster $A_{m}$ to its nearest *α* neighboring cluster centroids, which is computed as $\delta_{m,in}^{\mathrm{mutual}}=\left|v_{i}^{\vec{m}}-\vec{c_{n}}\right|$. Here, $\vec{v_{i}^{m}}\in A_{m}$, and $\vec{c_{n}}\in \xi_{m,\alpha }$ where $\xi_{m,\alpha }$ is the set of *α* nearest neighbors of $\vec{c_{m}}$. Therefore, $\delta_{m,in}^{\mathrm{mutual}}$ is a $\left|A_{m}\right|$-by-*α* asymmetrical matrix.

The above-mentioned approach provides computations of distance matrices from the feature matrix $\left\{{\vec{v}}_{i}\right\}$. In some cases, such as computing path length in a graph, the feature matrix is unknown, and the relation between samples was characterized directly by the distance matrix. Therefore, we also provide an approach to estimate the $\delta_{mn}$ and $\delta_{m,in}^{\mathrm{mutual}}$ from the original distance matrix (Supplementary).

#### Global embedding.

Global embedding performs BHMDS on $\delta_{mn}$ and gets low-dimensional representations of cluster centroids in *D*-dimensional hyperbolic space $H^{D}$. Denote the optimized cluster centroids in $H^{D}$ as $\vec{y_{k}}$.

#### Local embedding.

For each cluster *m*, local embedding is performed with a modified version of the BHMDS optimization (the “Relax” optimization): given a set of existing fixed locations $\{\vec{y_{n}}\}\in H^{D}$, embed a new set of samples optimizing over only their distance matrix $\delta_{m,ij}^{\mathrm{self}}$ and their distances to existing points $\delta_{m,in}^{\mathrm{mutual}}$. Therefore, instead of summing over all sample pairs (*i*, *j*) in eq. 5, the summation term in the new objective function is approximated by two terms: $\sum_{i,j}\to (\sum_{im,jm}+\sum_{im,n})$. The first term is a summation of distances between new samples (in this case, samples in $A_{m}$), and the second term is a summation of distances between new samples and existing points. It is worth noticing that all the cluster centroid locations needed for local embedding have been computed in the first step, which means local embedding can be parallelized across clusters.

#### Integrate embedding results.

From each local embedding of cluster *k*, the optimized locations of individual samples in $H^{D}$ were obtained as $\left\{\vec{x_{i}^{k}}\right\}$. Combining all embedding results, $\cup \{\vec{x_{i}^{k}}\}$ gives the final embedding positions of all samples $\vec{v_{i}}$ in $H^{D}$.

#### Map outliers.

When the cluster size and number control procedure is applied, clusters smaller than a specified threshold are excluded from both the global and local embedding steps. Once the embedding results for the remaining data points have been integrated, the previously excluded outliers are remapped into the embedding space using an approach similar to the local embedding step. Given the embeddings $\left\{\vec{x_{j}}\right\}$, each “outlier” $\vec{v_{i}}$ is mapped to the space based on its distance to its nearest neighbors, $\delta_{i,j}^{\mathrm{mutual}}$, $j\in \xi_{i,\alpha }$. After this step, the embedded outliers are integrated with the previously embedded points, resulting in the final embedding of all samples.

### Proof of the dissimilarity matrix approximation in MuH-MDS algorithm

The global-local embedding procedure provides an effective approximation to the original distance matrix $\delta_{ij}$. Denote the actual distance being optimized in global-local approach as $\delta_{ij}^{\prime }$. Consider the following three types of sample pairs: (1) sample pair within cluster; (2) sample pair between nearest neighbor clusters; and (3) sample pair between clusters that are not nearest neighbor clusters.

#### Within cluster.

By definition of local embedding process, distance within cluster is exactly optimized: $\delta_{ij}^{\prime }=\delta_{k,ij}^{\mathrm{self}}=\delta_{ij}$.

#### Between nearest neighbor clusters.

By definition of local embedding process, the distance $\delta_{ij}$ between samples belonging to nearest neighbor clusters was approximated as $\delta_{m,in}^{\mathrm{mutual}}$, the distance between sample $v_{i}^{\vec{m}}$ and the cluster centroid $\vec{c_{n}}$ which $\vec{v_{j}^{n}}$ belongs to. Denote $\vec{\Delta_{ij}^{\prime }}\equiv \vec{v_{i}^{m}}-\vec{c_{n}}$, then $\delta_{ij}^{\prime }=\delta_{m,in}^{\mathrm{mutual}}=\left|\Delta_{ij}^{\prime }\right|$.

Given that the samples $\left\{\vec{v_{i}}\right\}$ are grouped into clusters $\left\{A_{k}\right\}$, $\vec{v_{i}^{k}}$ could be written as a displacement from the cluster centroid that it belongs: $\vec{v_{i}^{k}}=\vec{c_{k}}+\vec{\eta_{i}^{k}}$. The displacement within cluster is much smaller than the displacement between cluster centroids, giving $|\vec{\eta_{i}^{k}}|\ll |\vec{c_{k}}-\vec{c_{m}}|,\forall m$, ∀*m*. Therefore, the true distance $\delta_{ij}$ between a sample $v_{i}^{\vec{m}}$ to another sample $v_{j}^{\vec{n}}$ belonging to its nearest neighbor cluster $A_{n}$ is:

(6) $$\delta_{ij}=\left|v_{i}^{\vec{m}}-\vec{v_{j}^{n}}\right|\;\;=\left|\vec{v_{i}^{m}}-\left(\vec{c_{n}}+\vec{\eta_{j}^{n}}\right)\right|\;\;=\left|\vec{{\Delta^{\prime }}_{ij}}-\vec{\eta_{j}^{n}}\right|\;\;={\left(\delta_{ij}^{\prime 2}+{\left|\vec{\eta_{j}^{n}}\right|}^{2}-2\vec{{\Delta^{\prime }}_{ij}}\cdot \vec{\eta_{j}^{n}}\right)}^{\frac{1}{2}}$$

Since $\left|\vec{\eta_{i}^{k}}|\ll |\vec{c_{k}}-\vec{c_{m}}\right|$, we have $\delta_{ij}^{\prime }=|\vec{v_{i}^{m}}-\vec{c_{n}}|=|\vec{c_{m}}+\vec{\eta_{i}^{m}}-\vec{c_{n}}|=|\vec{c_{m}}-\vec{c_{n}}|+o(\left|\vec{\eta_{i}^{m}}\right|)\gg |\vec{\eta_{j}^{n}}|$. Therefore, $\delta_{ij}={\left(\delta_{ij}^{\prime 2}+{\left|{\vec{\eta }}_{j}^{n}\right|}^{2}-2\vec{\Delta_{ij}^{\prime }}\cdot \vec{\eta_{j}^{n}}\right)}^{\frac{1}{2}}={\left(\delta_{ij}^{\prime 2}+o(\left|\vec{\eta_{j}^{n}}\right|)\right)}^{\frac{1}{2}}\approx \delta_{ij}^{\prime }$.

#### Between clusters that are not nearest neighbor clusters.

In this case, the true distance $\delta_{ij}$ could be written as $\delta_{ij}=|\vec{c_{m}}-\vec{c_{n}}+\eta_{i}^{m}-\vec{\eta_{j}^{n}}|=|\vec{c_{m}}-\vec{c_{n}}|+o(\left|\vec{\eta_{i}^{k}}\right|)\approx \delta_{mn}$, based on similar arguments since that $\left|\vec{\eta_{i}^{k}}|\ll |\vec{c_{k}}-\vec{c_{m}}\right|$. On the other hand, considering the embedding space $H^{D}$ and embedded cluster centroids $\vec{y_{k}}$ and samples $\vec{x_{i}^{k}}$, the equivalent distance approximated by global-local process $\delta_{ij}^{\prime }$ is the distance between $x_{i}^{\vec{m}}$ and $x_{j}^{n}$. Leveraging the Poincaré disk model ^26^, we have $\delta_{ij}^{\prime }=d^{H}(u_{i}^{\vec{m}},\vec{u_{j}^{n}})$, where

(7) $$d^{H}(\vec{u},\vec{v})=\text{arccosh}\left(1+2\frac{|\vec{u}-\vec{v}{|}^{2}}{\left(1-|\vec{u}{|}^{2}\right)\left(1-|\vec{v}{|}^{2}\right)}\right)$$

is the distance and $\vec{u_{i}^{k}}$ is the representation of $\vec{x_{i}^{k}}$ in the Poincaré disk model. Similarly, we denote the representation of $\vec{y_{m}}$ in the Poincaré disk as $\vec{w_{m}}$. In the global embedding, $d^{H}(\vec{w_{m}},\vec{w_{n}})$ is directly optimized from $\delta_{mn}$, giving $d^{H}(\vec{w_{m}},\vec{w_{n}})\approx d_{mn}$. In the local embedding, $d^{H}(\vec{w_{m}},\vec{u_{i}^{m}})$ is directly optimized, given that $d^{H}(\vec{w_{m}},\vec{u_{i}^{m}})\approx |\vec{c_{m}}-\vec{v_{i}^{m}}|=|\vec{\eta_{i}^{m}}|\ll \delta_{mn}$. Therefore, $\vec{u_{i}^{m}}$ can also be written as a small perturbation to the centroid location: ${\vec{u}}_{i}^{m}=\vec{w_{m}}⊕\epsilon_{i}^{\vec{m}}$, where

(8) $$\vec{u}⊕\vec{v}=\frac{\left(1+2\vec{u}\cdot \vec{v}\;+\;|\vec{v}{|}^{2}\right)\vec{u}\;+\;\left(1-|\vec{u}{|}^{2}\right)\vec{v}}{1+2\vec{u}\cdot \vec{v}+|\vec{u}{|}^{2}|\vec{v}{|}^{2}}$$

is the Möbius addition, vector addition of points in the Poincaré ball model. Calculating eq. 8, we got $u_{i}^{m}=\vec{w_{m}}+o(\left|\epsilon_{i}^{m}\right|)$. Plugging this into eq. 7 gives: (Supplementary)

(9) $$d^{H}\left(\vec{u_{i}^{m}},\vec{u_{j}^{n}}\right)=\text{arccosh}\left(1+2\frac{{\left|\vec{w_{m}}-\vec{w_{n}}\right|}^{2}+o(\vec{\epsilon })}{\left(1-{\left|\vec{w_{m}}\right|}^{2}+o(\vec{\epsilon })\right)\left(1-{\left|\vec{w_{n}}\right|}^{2}+o(\vec{\epsilon })\right)}\right)$$

(10) $$\approx \text{arccosh}\left(1+2\frac{{\left|\vec{w_{m}}-\vec{w_{n}}\right|}^{2}}{\left(1-{\left|w_{m}\right|}^{2}\right)\left(1-{\left|\vec{w_{n}}\right|}^{2}\right)}\right)$$

(11) $$=d^{H}\left(\vec{w_{m}},\vec{w_{n}}\right)$$

Therefore, $\delta_{ij}^{\prime }\approx \delta_{mn}\approx \delta_{ij}$. The intuition behind this is that distance between samples belonging to non-nearest-neighbor clusters are not directly optimized. Instead, this distance is approximated by fixing positions of cluster centroids in the global step, and anchoring samples to their nearest clusters in the local step, assuming that local distance is sufficient to constrain the position of samples in low dimensional space.

### Computing time estimation

In the paper, all simulations were conducted on a single x86-64 CPU without parallelization.

Suppose there are n samples divided into *k* clusters, the average cluster size is $\frac{n}{k}$. Then the global computing time $t_{\mathrm{global}}∼O(k^{2})$.

#### Without parallelization.

When local embedding is not parallelized, local embedding time $t_{\mathrm{local}}∼O(k{\left(\frac{n}{k}\right)}^{2})∼O(\frac{n^{2}}{k})$.

When $k^{2}\geq \frac{n^{2}}{k}$, then $k\geq n^{2/3}$, computing time is dominated by $t_{\mathrm{global}}∼O(k^{2})$ with the minimum obtained at $k=n^{2/3}$, which is $O(n^{4/3})$.

When $k^{2}\leq \frac{n^{2}}{k}$, then $k\leq n^{2/3}$, computing time is dominated by $t_{\mathrm{local}}∼O(\frac{n^{2}}{k})$ with the minimum obtained at $k=n^{2/3}$, which is also $O(n^{4/3})$.

#### With parallelization.

When local embedding is parallelized, local embedding time $t_{\mathrm{local}}∼O({\left(\frac{n}{k}\right)}^{2})∼O(\frac{n^{2}}{k^{2}})$.

When $k^{2}\geq \frac{n^{2}}{k^{2}}$, then $k\geq n^{1/2}$, computing time is dominated by $t_{\mathrm{global}}∼O(k^{2})$ with the minimum obtained at $k=n^{1/2}$, which is *O*(*n*).

When $k^{2}\leq \frac{n^{2}}{k^{2}}$, then $k\leq n^{1/2}$, computing time is dominated by $t_{\mathrm{local}}∼O(\frac{n^{2}}{k^{2}})$ with the minimum obtained at $k=n^{1/2}$, which is also *O*(*n*).

### Dataset descriptions

#### scRNA-seq datasets.

For datasets with feature numbers larger than 100, we use PCA to reduce the dimension to 100 before applying the embedding, as well as other competing methods. This is widely used to denoise the data, given the sparsity of single-cell datasets.

#### Toggle Switch.

Synthetic data obtained by simulating a simple toggle switch ^20^ using Scanpy ^29^, a widely used large-scale single-cell gene expression data analysis tool. It contains 200 samples with 2 markers characterizing a differentiation process with two branches.

#### Myeloid Progenitors (Krumsiek11).

Synthetic data obtained from simulating differentiation of myeloid progenitors, using Scanpy^29^. It contains 640 samples with 11 markers representing cell differentiation of a common myeloid progenitor state to four cell fates: erythrocyte, neutrophil, monocyte, and megakaryocyte.

#### Mouse Myelopoesis (Olsson).

The mouse myelopoesis dataset ^21^ contains 382 samples belonging to 9 cell types: HSCP-1 (hematopoietic stem cell progenitor), HSCP-2, megakaryocytic, erythrocytic, Multi-Lin (multi-lineage primed), MDP (monocyte-dendritic cell precursor), monocytic, granulocytic and myelocyte (myelocytes and metamyelocytes). We downloaded preprocessed data from Klimovskaia et al.^3^, with 382 samples and 533 features.

#### Mouse myeloid progenitors dataset (Paul).

The mouse myeloid progenitors dataset is a MARS-seq dataset from Paul et al. ^22^. We loaded from Scanpy and preprocessed using the pipeline offered by Scanpy (sc.pp.recipe zheng17()). After preprocessing, there are 2730 samples and 999 genes kept. The original paper identified 19 clusters.

#### *C. elegans* embryogenesis.

The *C. elegans* dataset is 10X Genomics data from Packer et al. ^23^. It contains transcript data of single cells from *C. elegans* embryos at developmental stages ranging from gastrulation to terminal cell differentiation, with 37 main cell types, and embryo time ranging from 0 to 830 minutes. The data is publicly available at GEO:GSE126954. The original dataset has 89701 samples and 20222 genes. We preprocessed it using a standard Scanpy pipeline for 10x genomics. After preprocessing, there are 85,333 samples with 2756 genes. Since competing methods like Poincaré map take a very long time to run for large datasets, we randomly sampled 10,000 and 40,000 samples from the full dataset to create smaller datasets for benchmarking. To analyze cell lineages, we create another subset only containing the ABpxp ectodermal lineage, where the descendants are labeled by attaching either “p”(posterior) or “a”(anterior) (ABpxpa, ABpxpp, ABpxpaa, ABpxpap, etc. See Fig. 4a). The ABpxp lineage dataset has 7,562 samples with 2,756 genes, divided into 72 different lineages.

#### WordNet.

WordNet^27^ is a large lexical database of English. We embedded the mammal subtree of WordNet to demonstrate that MuH-MDS can be generally applied to a variety of datasets. We took the graph data of the mammal subtree from Nickel and Kiela^7^ and computed the distance matrix based on the graph path length where each node is only connected to its direct ancestor.

#### Graph Datasets.

We evaluated our method on four graph datasets originally introduced by De Sa et al. ^30^ and obtained data from Chami et al. ^10^. These datasets represent diverse hierarchical structures and have been widely used in prior work on hyperbolic embedding. Specifically, they include: a fully balanced tree with 40 nodes, a phylogenetic tree with 344 nodes, a biological disease graph representing disease relationships, with 516 nodes, and a Computer Science Ph.D. advisor–advisee network containing 1025 nodes. We directly obtained the edge list files from the repository and constructed the undirected graph for each dataset. For embedding purposes, we computed the shortest path (graph geodesic) distance matrix between all pairs of nodes in each graph. This distance matrix was then used as input for our embedding algorithm.

### Competing Methods

We compared with competing methods. Details can be found in the code released.

#### Poincaré Map.

We compared with Poincaré Map using the code provided by the authors^3^ from GitHub. All datasets were simulated based on the example usage parameters.

#### Phate.

We compared with Phate^18^ using phate 1.0.11. Using default parameters, we embedded data into either 2 or 3 dimensions. The documentation of Phate can be found here: link.

#### Scanpy.

Results for the following competing methods: diffusion map^15^, UMAP^24^, T-sne^25^, PCA, ForceAtlas2^19^ were fitted using scanpy (scanpy 1.9.8), a single cell analysis tool^29^. T-sne and ForceAtlas2 are only in 2-dimension, and other methods are in either 2- or 3-dimension. Scanpy Documentation: link.

### Embedding quality metrics

To quantify the embedding performance, we computed a scale independent quality criteria proposed by Lee at al. ^31^ The intuition is to compute a co-ranking matrix from ranks of distance in original high dimensional space and ranks of distance in the embedded low dimensional space. Klimovskaia et al. presented detailed calculation of $Q_{\mathrm{local}}$ and $Q_{\mathrm{global}}$ and publicly available code.

In WordNet embedding, we computed the distortion rate using the following formula^10^:

$$\frac{1}{\left(\begin{array}{c} \left|S\right|\\ 2 \end{array}\right)}\underset{x\neq y\in S}{\sum }\frac{\left|d^{H}(\pi (x),\pi (y))-d(x,y)\right|}{d(x,y)}$$

where $d^{H}(a,b)$ is the hyperbolic distance between $a,b\in H^{D},\pi (x)$ is the hyperbolic embedding of *x*, and d(x,y) is the original distance.

### Cell lineage data analysis

#### Logarithmic map.

Logarithmic map maps a point *x* in the hyperbolic space to a vector in the tangent plane of a given point *p* such that the vector norm of *log*(*x*) is equal the distance from *p* to *x*. The mapping to tangent space helps generalize matrix and vector calculations into hyperbolic space^26^. For *p* at the origin, so $p^{\alpha }=(1,0,0,\ldots ,0)$, the logarithmic map of $x^{\alpha }$ is given by

(12) $${\text{log}}_{p}\left(x^{\alpha }\right)=\text{arcosh}\left(x_{0}\right)\frac{x^{\alpha }-x_{0}p^{\alpha }}{\sqrt{\vec{x}\cdot \vec{x}}}$$

To visualize lineages embedded in 7-dimensional hyperbolic space, we use logarithmic map to project them onto 2-d tangent space. In practice, we used another valid form in Poincaré disk to calculate the vector in tangent space^26^: ${log}_{0}^{c}(y)=arctanh(|y|)\frac{y}{\left|y\right|}$. In tangent space, Euclidean vector and matrix computations could be performed.

#### Fitting projection axis.

To evaluate how well the learned representation preserved biologically meaningful axes, we performed repeated correlation-based projection analysis. Specifically, for each of 20 repetitions, samples were randomly split into 50% training and 50% test sets. In each split, the training set was used to fit a one-dimensional projection direction that maximized correlation with either generation depth or anterior–posterior (A–P) position, using L-BFGS-B optimization in Euclidean space. The learned projection was then applied to the held-out test set, and the correlation between projected coordinates and the corresponding ground truth labels was computed for evaluation.

#### Diffusion pseudotime (DPT).

DPT was computed using the sc.tl.diffmap and sc.tl.dpt functions in Scanpy^29^. For each lineage, cells were embedded into a diffusion map constructed from a k-nearest neighbor graph (with k=10) based on the input representation. The root cell was selected as the one closest to the centroid of a designated early lineage population, and pseudotime values were then inferred from diffusion components.

## Supplementary Material

1

## Data Availability

The code used in this study will be made publicly available on GitHub upon publication.
