# Supplementary material for: Multiscale Hyperbolic Embedding for Cell Hierarchies in Large-Scale Bioinformatics Data
Source: bioRxiv. 2025 Oct 1:2025.09.29.679407. Preprint. [Version 1] doi: 10.1101/2025.09.29.679407 (PMC12621726; doi:10.1101/2025.09.29.679407)
Supplement: 1 [file NIHPP2025.09.29.679407v1-supplement-1.pdf]

bioRxiv preprint doi: https://doi.org/10.1101/2025.09.29.679407; this version posted October 1, 2025. The copyright holder for this preprint (which was not certified by peer review) is the author/funder, who has granted bioRxiv a license to display the preprint in perpetuity. It is made available under aCC-BY 4.0 International license.

Supplementary materials

Pseudo code for MuH-MDS

Algorithm 1 shows the M-HMDS algorithm. In line 6, δ_{ij} is a pairwise distance matrix of samples in cluster m and cluster n; $MInd$ and $NInd$ indicates which samples belongs to cluster m or n. In line 13, $d_{i,\{n\}}$ is a pairwise distance matrix of sample i combined with samples in cluster n. In line 19, $\{\vec{y_k}\}_{NNs}$ is set of locations of cluster centroids of the nearest neighbors of cluster m. In line 25, $\{\vec{x_p}\}_{nns}$ is locations of nearest neighboring points of the outlier $\{\vec{x_p}\}$, and $d^{mutual}_{p,\{\vec{x_p}\}_{nns}}$ is mutual distance from outliers to them.

bioRxiv preprint doi: https://doi.org/10.1101/2025.09.29.679407; this version posted October 1, 2025. The copyright holder for this preprint (which was not certified by peer review) is the author/funder, who has granted bioRxiv a license to display the preprint in perpetuity. It is made available under aCC-BY 4.0 International license.

Algorithm 1 Multiscale hyperbolic multidimensional scaling (MuH-MDS)

Input Sample feature matrix $\{\vec{v_i}\}$ or pairwise distance matrix δ_{ij}, number of nearest neighbors α, number of hyperbolic space dimension D, maximal/minimal number of samples in each cluster $[n_{max}, n_{min}]$, Boolean variable $MapOutier$ determining whether to map outliers
Output Embedding coordinates in $\mathbb{H}^D$: $\{\vec{x_i}\}$
$ClusterIDs, OutlierIDs \leftarrow$ ClusteringAndFiltering($\{\vec{v_i}\}$ or d_{ij}, n_{max}, n_{min})
for m, n in Unique($ClusterIDs$) **do**
    $\delta_{mn} \leftarrow$ GetDmatFeature($\{\vec{v}\}_m$,$\{\vec{v}\}_n$)
    or GetDmatDistance($\delta_{ij}, MInd, NInd$)
end for
for m in Unique($ClusterIDs$) **do**
    $NNs \leftarrow \alpha$ nearest neighbors of cluster m
    for i in cluster m **do**
        for n in NNs **do**
            $\delta^{mutual}_{m,in} \leftarrow$ GetDmatFeature($\vec{v_i}$, $\{\vec{v}\}_n$)
            or GetDmatDistance($\delta_{i,\{n\}}, MInd = 0, NInd = [1, 2, ...len(d) - 1]$)
        end for
    end for
end for
$\{\vec{y_k}\} \leftarrow$ HMDS(δ_{mn})
for m in Unique($ClusterIDs$) **do**
    (In parallel) $\{x_i^{\vec{m}}\} \leftarrow$ Relax($\delta^{mutual}_{m,in}$, $\delta^{self}_{k,ij}$, $\{\vec{y_k}\}_{NNs}$)
end for
(comment: *HMDS* algorithm were described in the main text and BHMDS paper[12], *Relax* algorithm were described in Methods)
$\{\vec{x_i}\} \leftarrow \cup\{x_i^{\vec{m}}\}$
if $MapOutier$ **then**
    for p in Outliers **do**
        $\vec{x_p} \leftarrow$ Relax($d^{mutual}_{p,\{\vec{x_p}\}_{nns}}$,$\{\vec{x}_{p_{nns}}\}$)
        $\{\vec{x_i}\} \leftarrow \{\vec{x_i}\} \cup \{\vec{x_p}\}$
    end for
end if
return $\{\vec{x_i}\}$

Algorithm 2 simply defines the function for clustering, filtering and cluster size control.

bioRxiv preprint doi: https://doi.org/10.1101/2025.09.29.679407; this version posted October 1, 2025. The copyright holder for this preprint (which was not certified by peer review) is the author/funder, who has granted bioRxiv a license to display the preprint in perpetuity. It is made available under aCC-BY 4.0 International license.

Algorithm 2 ClusteringAndFiltering($\{\vec{v_i}\}$ or $\delta_{ij}, n_{max}, n_{min}$)

Input Sample feature matrix $\{\vec{v_i}\}$ or pairwise distance matrix δ_{ij}, maximal/minimal number of samples in each cluster $[n_{max}, n_{min}]$
Output Assignment of cluster for each sample $ClusterIDs$
$ClusterIDs \leftarrow$ Cluster assignment of each sample, from clustering on feature matrix $\{\vec{v_i}\}$ or pairwise distance matrix δ_{ij}
$ClusterIDs, OutlierIDs \leftarrow$ Remove clusters whose size is smaller than n_{min}, re-divide clusters whose size larger than n_{max} until their size is no greater than n_{max}
return $ClusterIDs, OutlierIDs$

Compute distance to clusters from distance matrix

Algorithm 3 and 4 defines two different functions to fill the needs for different input types: feature matrix or pair-wise distance matrix. In Algorithm 1, it will determine which distance computing function to implement depend on the input type.

Algorithm 3 GetDmatFeature($\{\vec{u}\}, \{\vec{v}\}$)

Input Two set of vectors from the same vector space $\{\vec{u}\}, \{\vec{v}\}$
Output d, Average distance between the two sets
$d \leftarrow |$Mean($\{\vec{u}\}$)-Mean($\{\vec{v}\}$)$|$
return d

Algorithm 4 GetDmatDistance

Input Distance matrix D, two sets of unique index $I = \{i\}$ and $J = \{j\}$ with $I \cap J = \{\}$, and $|I \cup J|$=len(D). I and J indicate which points belongs to which of the two clusters.
Output d, Average distance between the two sets
for j in J **do**
    $d_{Ij} \leftarrow \text{sqrt}(\frac{1}{|I|}\sum_{i \in I} d_{ij} - \frac{1}{|I|^2}\sum_{i,i' \in I, i' < i} d_{i,i'})$
end for
$d \leftarrow \text{sqrt}(\frac{1}{|J|}\sum_{j \in J} d_{Ij} - \frac{1}{|J|^2}\sum_{j,j' \in J, j' < j} d_{j,j'})$
return d

The computation in Algorithm 4 is based on following approximations: Suppose we have a set of n vectors $\vec{y_i}$ and a vector $\vec{x}$. Let $\vec{d_i} = \vec{x} - \vec{y_i}$. The goal is to approximate $d = |\vec{x} - Mean(\vec{y_i})|$ from $d_i \equiv |\vec{x} - \vec{y_i}| = |\vec{d_i}|$ and $d_{ii'} \equiv |\vec{y_i} - \vec{y_{i'}}| = |\vec{d_i} - \vec{d_{i'}}|$.

bioRxiv preprint doi: https://doi.org/10.1101/2025.09.29.679407; this version posted October 1, 2025. The copyright holder for this preprint (which was not certified by peer review) is the author/funder, who has granted bioRxiv a license to display the preprint in perpetuity. It is made available under aCC-BY 4.0 International license.

We have

$$\begin{aligned} d &= |\vec{x} - \frac{1}{n}\sum_n \vec{y_i}| = \frac{1}{n}|\sum_n (\vec{x} - \vec{y_i})| = \frac{1}{n}|\sum_n (\vec{d_i})| \\ &= \frac{1}{n}\sqrt{\sum_i d_i^2 + 2\sum_{i<i'} \vec{d_i}\cdot\vec{d_{i'}}} \end{aligned}$$

Since $d_{ii'}^2 = |\vec{d_i} - \vec{d_{i'}}|^2 = d_i^2 + d_{i'}^2 - 2\vec{d_i}\cdot\vec{d_{i'}}$, we have $2\vec{d_i}\cdot\vec{d_{i'}} = d_i^2 + d_{i'}^2 - d_{ii'}^2$. Therefore:

$$\begin{aligned} d &= \frac{1}{n}\sqrt{\sum_i d_i^2 + \sum_{i<i'} d_i^2 + d_{i'}^2 - d_{ii'}^2} \\ &= \frac{1}{n}\sqrt{n\sum_i d_i^2 - \sum_{i<i'} d_{ii'}^2} \\ &= \sqrt{\frac{1}{n}\sum_i d_i^2 - \frac{1}{n^2}\sum_{i<i'} d_{ii'}^2} \end{aligned}$$

One might noticed that Algorithm 4 depends on the order of performing summation on different clusters. We set the order by the variance of original local distance matrix, and sum over cluster with smaller variance first.

Approximation on distance between clusters that are not nearest neighbor clusters

Eq. 8 gives the Möbius addition:

$$\vec{u} \oplus \vec{v} = \frac{(1 + 2\vec{u}\cdot\vec{v} + |\vec{v}|^2)\vec{u} + (1 - |\vec{u}|^2)\vec{v}}{1 + 2\vec{u}\cdot\vec{v} + |\vec{u}|^2|\vec{v}|^2}$$

bioRxiv preprint doi: https://doi.org/10.1101/2025.09.29.679407; this version posted October 1, 2025. The copyright holder for this preprint (which was not certified by peer review) is the author/funder, who has granted bioRxiv a license to display the preprint in perpetuity. It is made available under aCC-BY 4.0 International license.

Therefore,

$$\begin{aligned} \vec{u_i^m} &= \vec{w_m} \oplus \vec{\epsilon_i^m} \\ &= \frac{(1 + 2\vec{w_m} \cdot \vec{\epsilon_i^m} + |\vec{\epsilon_i^m}|^2)\vec{w_m} + (1 - |\vec{w_m}|^2)\vec{\epsilon_i^m}}{1 + 2\vec{w_m} \cdot \vec{\epsilon_i^m} + |\vec{w_m}|^2|\vec{\epsilon_i^m}|^2} \\ &= \frac{(1 + 2\vec{w_m} \cdot \vec{\epsilon_i^m} + o(|\vec{\epsilon}|^2))\vec{w_m} + (1 - |\vec{w_m}|^2)\vec{\epsilon_i^m}}{1 + 2\vec{w_m} \cdot \vec{\epsilon_i^m} + o(|\vec{\epsilon}|^2)} \\ &\approx \frac{(1 + 2\vec{w_m} \cdot \vec{\epsilon_i^m})\vec{w_m} + (1 - |\vec{w_m}|^2)\vec{\epsilon_i^m}}{1 + 2\vec{w_m} \cdot \vec{\epsilon_i^m}} \\ &= \vec{w_m} + \frac{1 - |\vec{w_m}|^2}{1 + 2\vec{w_m} \cdot \vec{\epsilon_i^m}}\vec{\epsilon_i^m} \end{aligned}$$

Let $\alpha_i^m = \frac{1-|\vec{w_m}|^2}{1+2\vec{w_m}\cdot\vec{\epsilon_i^m}}$, then $\vec{u_i^m} = \vec{w_m} + \alpha_i^m \vec{\epsilon_i^m}$, upon dropping the $o(\epsilon^2)$ term.

Consider the scale of α_i^m: Since $\vec{w_m} \cdot \vec{\epsilon_i^m} \geq -|\vec{w_m}||\vec{\epsilon_i^m}|$, we have $\alpha_i^m = \frac{1-|\vec{w_m}|^2}{1+2\vec{w_m}\cdot\vec{\epsilon_i^m}} \leq \frac{1-|\vec{w_m}|^2}{1-2|\vec{w_m}||\vec{\epsilon_i^m}|}$. Given that $|\vec{\epsilon_i^m}| \ll |\vec{w_m}|$, so $2|\vec{\epsilon_i^m}| \ll |\vec{w_m}|$, we have $\alpha_i^m \leq \frac{1-|\vec{w_m}|^2}{1-2|\vec{w_m}||\vec{\epsilon_i^m}|} = \frac{1-|\vec{w_m}|^2}{1-|\vec{w_m}|\cdot 2|\vec{\epsilon_i^m}|} \ll \frac{1-|\vec{w_m}|^2}{1-|\vec{w_m}|\cdot|\vec{w_m}|} = 1$.

Since $\vec{u_i^m} = \vec{w_m} + \alpha_i^m \vec{\epsilon_i^m}$ and $\alpha_i^m \ll 1$, we have $\vec{u_i^m} = \vec{w_m} + o(|\vec{\epsilon_i^m}|)$.

Eq. 7 gives the distance in Poincaré disk model:

$$d^H(\vec{u}, \vec{v}) = \text{arccosh}(1 + 2\frac{|\vec{u} - \vec{v}|^2}{(1 - |\vec{u}|^2)(1 - |\vec{v}|^2)})$$

Therefore,

$$\begin{aligned} d^H(\vec{u_i^m}, \vec{u_j^n}) &= d^H(\vec{w_m} + o(\epsilon), \vec{w_n} + o(\epsilon)) \\ &= \text{arccosh}(1 + 2\frac{|(\vec{w_m} + o(\epsilon)) - (\vec{w_n} + o(\epsilon))|^2}{(1 - |(\vec{w_m} + o(\epsilon))|^2)(1 - |(\vec{w_n} + o(\epsilon))|^2)}) \\ &= \text{arccosh}(1 + 2\frac{|\vec{w_m} - \vec{w_n}|^2 + o(\epsilon)}{(1 - |\vec{w_m}|^2 + o(\epsilon))(1 - |\vec{w_n}|^2 + o(\epsilon))}) \\ &\approx \text{arccosh}(1 + 2\frac{|\vec{w_m} - \vec{w_n}|^2}{(1 - |\vec{w_m}|^2)(1 - |\vec{w_n}|^2)}) \\ &= d^H(\vec{w_m}, \vec{w_n}) \end{aligned}$$

bioRxiv preprint doi: https://doi.org/10.1101/2025.09.29.679407; this version posted October 1, 2025. The copyright holder for this preprint (which was not certified by peer review) is the author/funder, who has granted bioRxiv a license to display the preprint in perpetuity. It is made available under aCC-BY 4.0 International license.

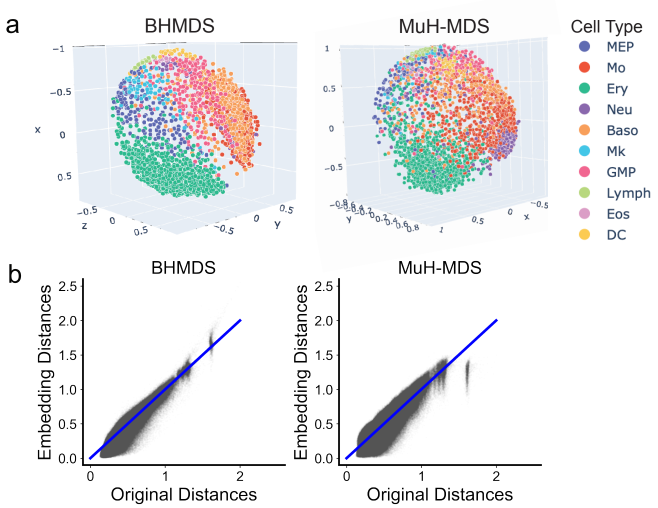

c

Metrics		BHMDS	MuH-MDS
Embedding Quality Metrics	Q-local	0.518583	0.414741
	Q-global	0.879617	0.826342
	Correlation	0.943675	0.855718
Hyperbolic Property	Curvature	7.947883	8.09052
Efficiency	Computing time (minutes)	**13806**	**15.00**

Figure S1: Comparison between the original (BHMDS) and multiscale (MuH-MDS) approaches using the mouse myeloid progenitors scRNA-seq dataset ("Paul")[22]. (a) Shepard diagram comparing original pairwise distances with embedded distances. (b) Embedding in 3-d hyperbolic space of "Paul" dataset. (c) Embedding metrics. MuH-MDS parameters: $\alpha = 30$, $k = 210$.

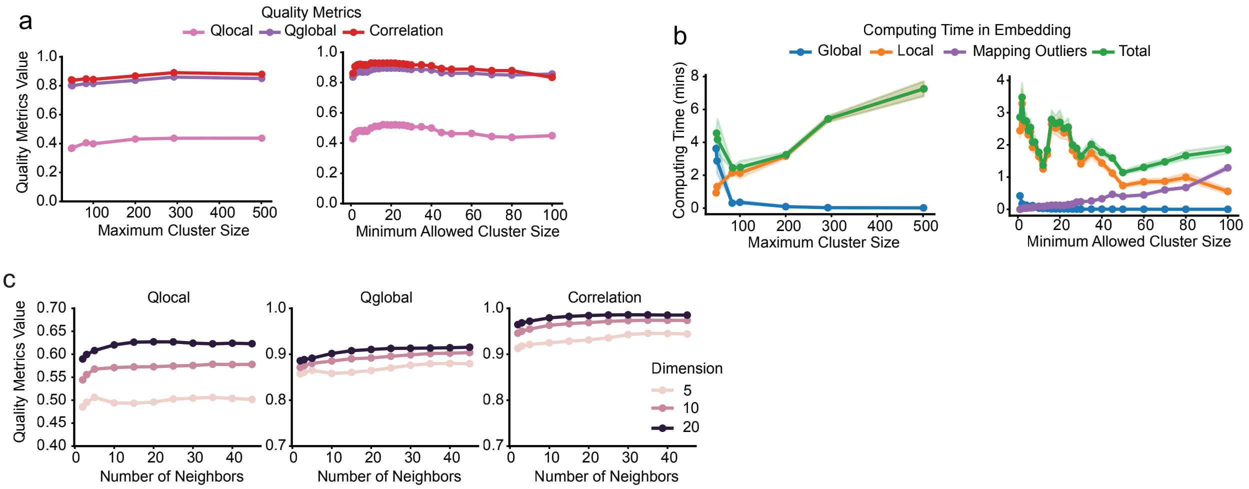

Figure S2: Simulation of hyperparameters using the Paul dataset[22], with 20 repetitions for each parameter setting. (a-b) Changes in embedding quality metrics (a) and running time (b) with varying maximum (left) or minimum (right) population sizes. (c) Embedding quality metrics across different numbers of nearest neighbors (α) in dimensions 5, 10, and 20. MuH-MDS parameters: (1) For maximum cluster size simulations: $k = 25$ $\alpha = 20$ $D = 3$; (2) For minimum allowed cluster size simulations: $k = 100$ $\alpha = 20$ $D = 3$; (3) For α and D simulations: $k = 50$.

bioRxiv preprint doi: https://doi.org/10.1101/2025.09.29.679407; this version posted October 1, 2025. The copyright holder for this preprint (which was not certified by peer review) is the author/funder, who has granted bioRxiv a license to display the preprint in perpetuity. It is made available under aCC-BY 4.0 International license.

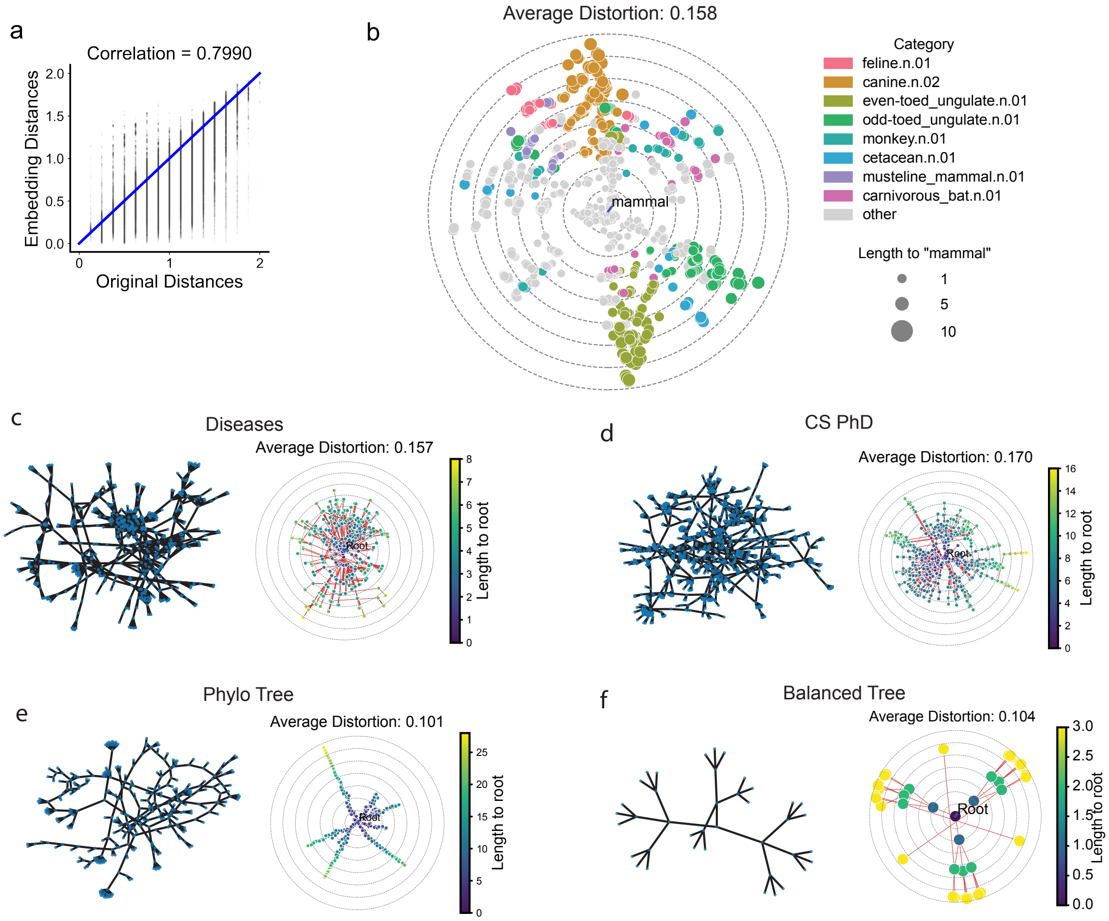

Figure S3: (a) Shepard diagram of WordNet mammal subtree embedding in 2-d hyperbolic space. (b) Application of MuH-MDS to the WordNet mammal subtree, shown in native coordinates. Marker size reflects the graph distance to the root node "mammal", while color denotes membership in semantic categories as shown in the legend. These categories correspond to parent nodes with the largest numbers of child nodes and were selected to highlight major structural branches within the hierarchy. MuH-MDS parameters: $k = 198$, $\alpha = 60$. (c-f) Embedding of multiple graph datasets from De Sa et al.[30] using MuH-MDS into 2D hyperbolic space. Left: original graph representations. Right: embedding results in 2D hyperbolic space. Color indicates graph distance to the root. Root is defined as the node with the smallest average distance to all other nodes in the original graph. MuH-MDS parameters: Diseases: $k = 106, \alpha = 60$, CS PhD: $k = 75, \alpha = 60$, Phylo Tree: $k = 34, \alpha = 20$, Balanced Tree: $k = 40, \alpha = 20$.

bioRxiv preprint doi: https://doi.org/10.1101/2025.09.29.679407; this version posted October 1, 2025. The copyright holder for this preprint (which was not certified by peer review) is the author/funder, who has granted bioRxiv a license to display the preprint in perpetuity. It is made available under aCC-BY 4.0 International license.

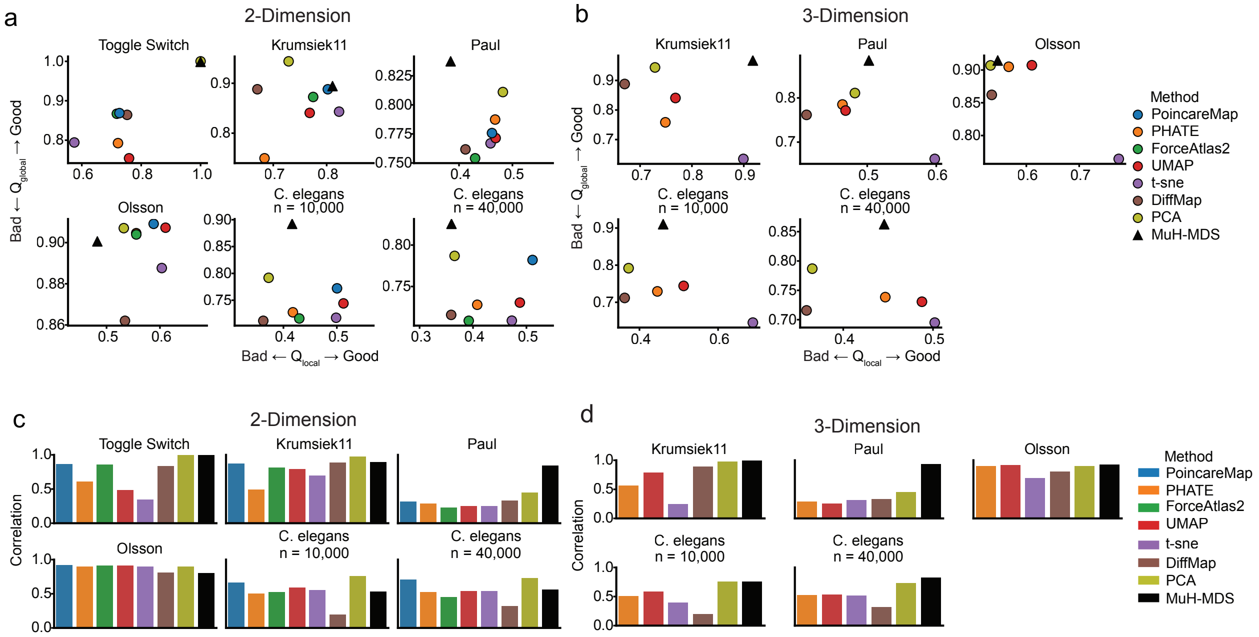

Figure S4: Embedding quality metric for all datasets tested in the paper, in 2-dimension (a-b) or 3-dimension (c-d) space. MuH-MDS parameters: Toggle switch, 2d: $\alpha = 10, k = 20$. Krumsiek11, 2d: $\alpha = 10, k = 50$; 3d: $\alpha = 20, k = 50$. Paul, 2d and 3d: $\alpha = 20, k = 100$. Olsson, 2d: $\alpha = 30, k = 40$; 3d: $\alpha = 20, k = 40$. *C. elegans* (10,000) , 2d: $\alpha = 20, k = 300$; 3d: $\alpha = 30, k = 300$. *C. elegans* (40,000) , 2d and 3d: $\alpha = 30, k = 200$. (α: number of neighbors, k: number of clusters)

bioRxiv preprint doi: https://doi.org/10.1101/2025.09.29.679407; this version posted October 1, 2025. The copyright holder for this preprint (which was not certified by peer review) is the author/funder, who has granted bioRxiv a license to display the preprint in perpetuity. It is made available under aCC-BY 4.0 International license.

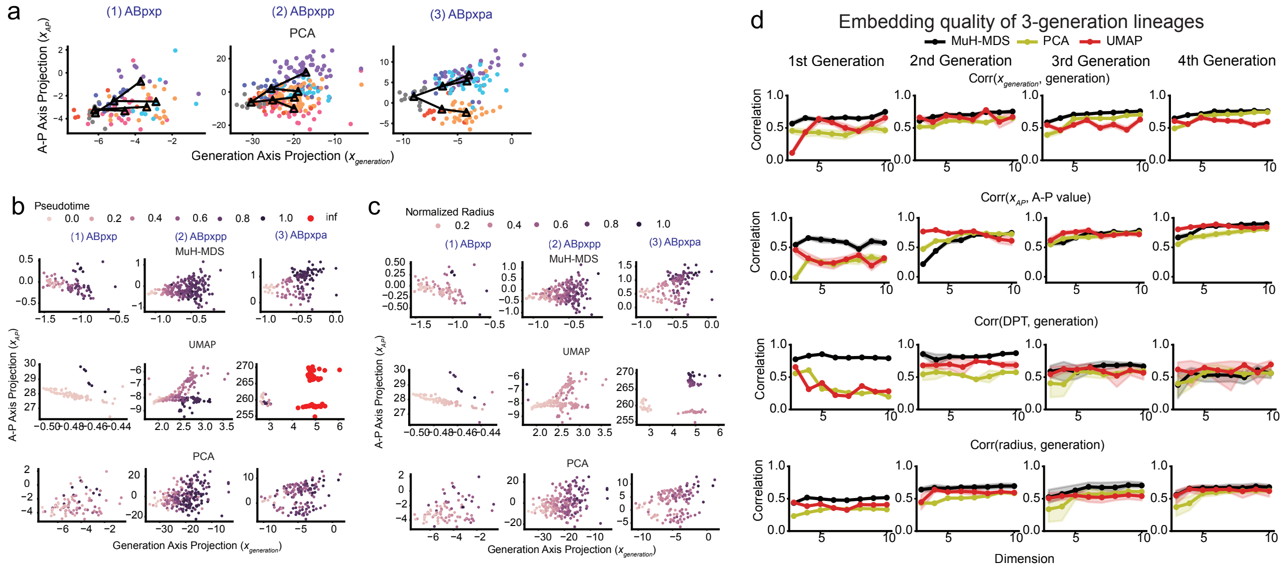

Figure S5: Analysis of the ABpxp lineage (7,562 samples) of *C. elegans* dataset. (a) 7-d PCA projecting to 2-d space, for 3-generation lineages starting from ABpxp, ABpxpp and ABpxpa. (b) Pseudotime computed from 7-d embedding of MuH-MDS, UMAP and PCA, for 3-generation lineages starting from ABpxp, ABpxpp and ABpxpa respectively. Some samples have infinite pseudotime because graph could be disconnected in high dimensional UMAP. (c) Normalize, re-centered radius computed from same embeddings as in (b). All embeddings are re-centered so that its population centroid is at the origin before computing the radius. (d) Comparison of 4 sub-lineage embedding quality metrics: (1) correlation between generation and projected coordinates (Corr($x_{\text{generation}}$, generation)), (2) correlation between A-P value and projected coordinates (Corr(x_{AP}, A-P value)), (3) correlation between generation and diffusion pseudotime inferred from embedding (Corr(DPT, generation)) and (4) correlation between generation and sample radius in embeddings (Corr(radius, generation)), across different dimensions, embedding methods (MuH-MDS, PCA, and UMAP), for lineages starting from 1st generation to 4th generation.

bioRxiv preprint doi: https://doi.org/10.1101/2025.09.29.679407; this version posted October 1, 2025. The copyright holder for this preprint (which was not certified by peer review) is the author/funder, who has granted bioRxiv a license to display the preprint in perpetuity. It is made available under aCC-BY 4.0 International license.

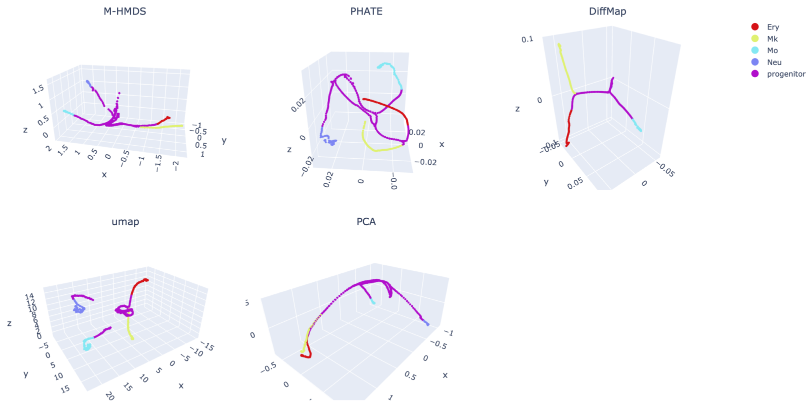

Figure S6: Embedding of the (synthetic) myeloid progenitors dataset (Krumsiek11), in 3-d space.

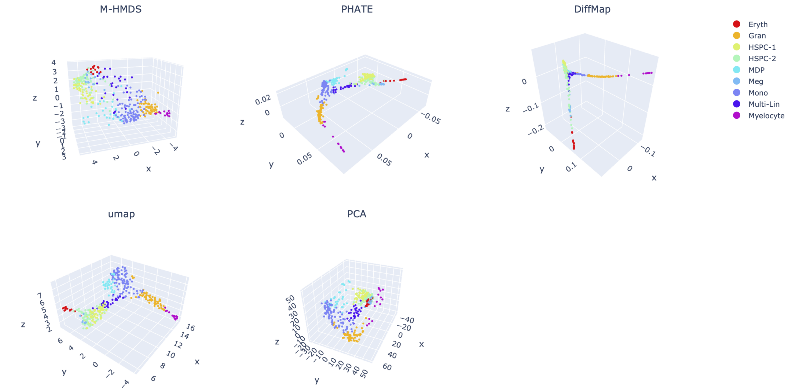

Figure S7: Embedding of the mouse myelopoesis dataset (Olsson), in 3-d space.

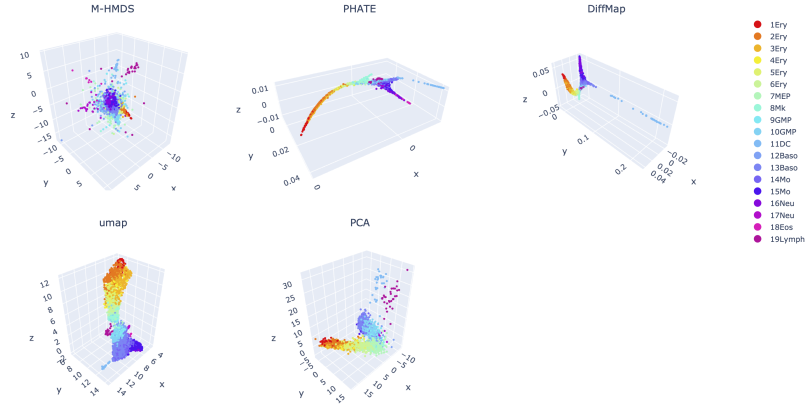

Figure S8: Embedding of the mouse myeloid progenitors dataset (Paul), in 3-d space.

bioRxiv preprint doi: https://doi.org/10.1101/2025.09.29.679407; this version posted October 1, 2025. The copyright holder for this preprint (which was not certified by peer review) is the author/funder, who has granted bioRxiv a license to display the preprint in perpetuity. It is made available under aCC-BY 4.0 International license.

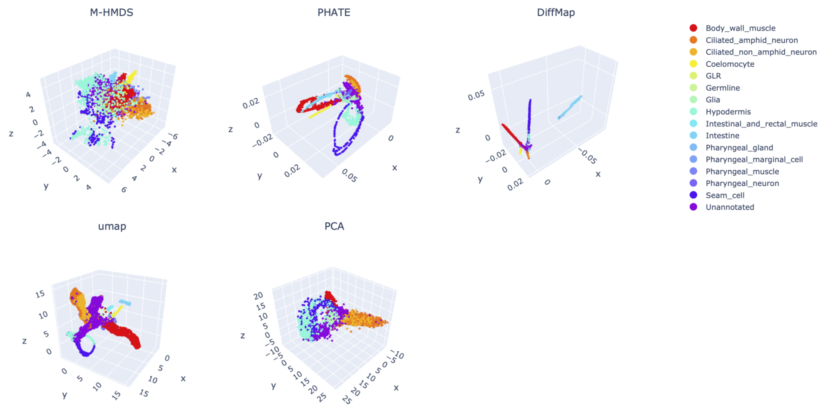

Figure S9: Embedding of the 10,000 sample subset of *C. elegans* dataset, in 3-d space, colored by cell types.

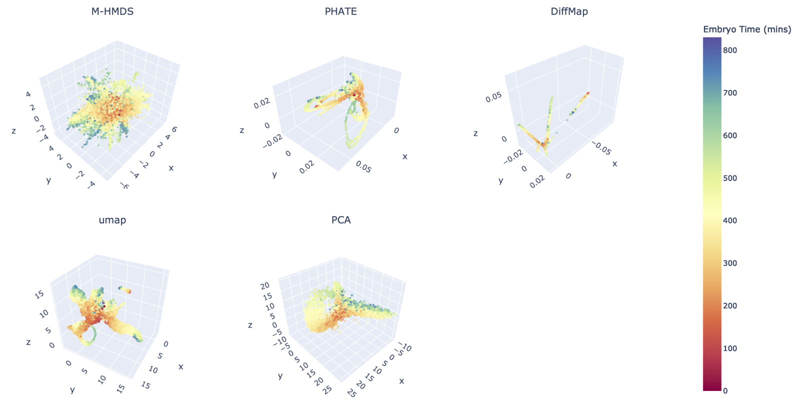

Figure S10: Same as Fig. (S9), but colored by embryo time.

bioRxiv preprint doi: https://doi.org/10.1101/2025.09.29.679407; this version posted October 1, 2025. The copyright holder for this preprint (which was not certified by peer review) is the author/funder, who has granted bioRxiv a license to display the preprint in perpetuity. It is made available under aCC-BY 4.0 International license.

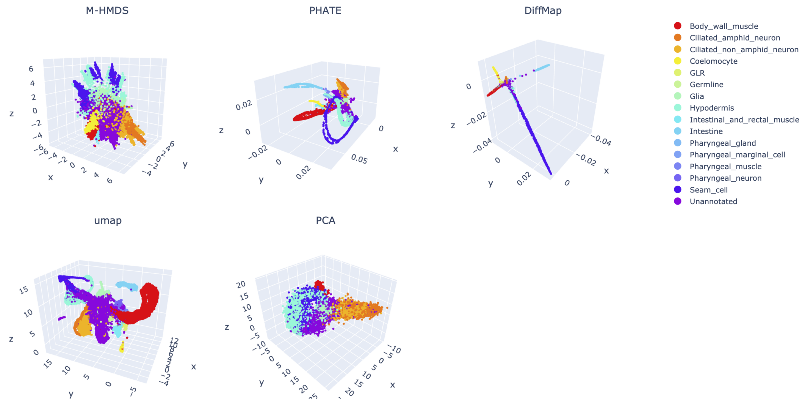

Figure S11: Embedding of the 40,000 sample subset of *C. elegans* dataset, in 3-d space, colored by cell types.

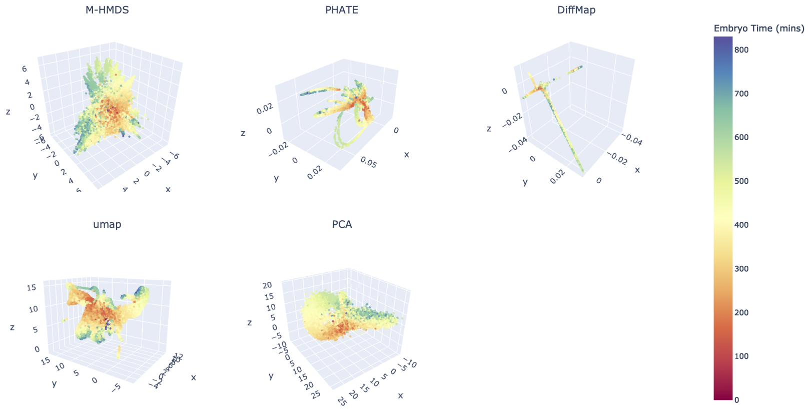

Figure S12: Same as Fig. (S11), but colored by embryo time.

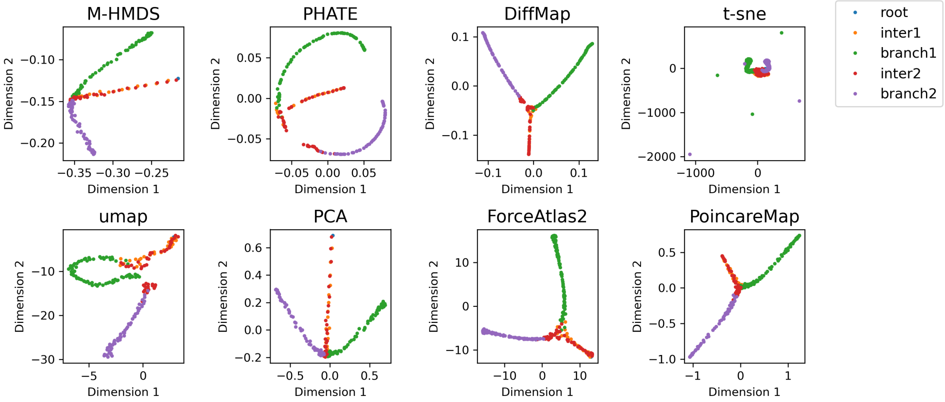

Figure S13: Embedding of the (synthetic) toggle switch dataset, in 2-d space.

bioRxiv preprint doi: https://doi.org/10.1101/2025.09.29.679407; this version posted October 1, 2025. The copyright holder for this preprint (which was not certified by peer review) is the author/funder, who has granted bioRxiv a license to display the preprint in perpetuity. It is made available under aCC-BY 4.0 International license.

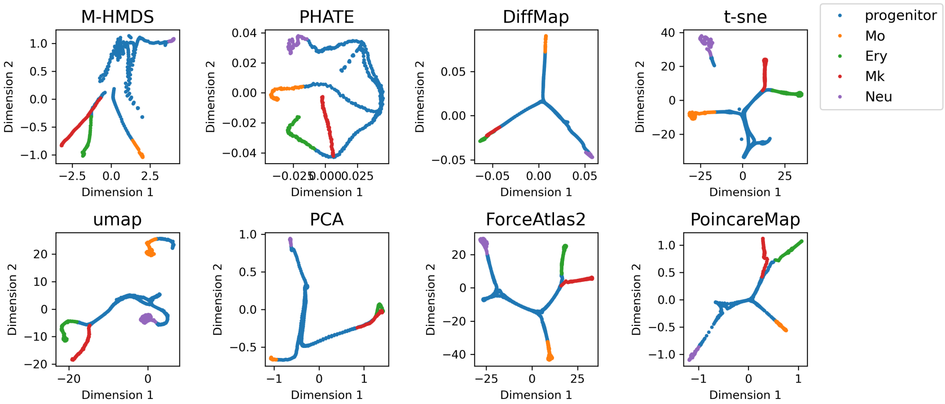

Figure S14: Embedding of the (synthetic) myeloid progenitors dataset (Krumsiek11), in 2-d space.

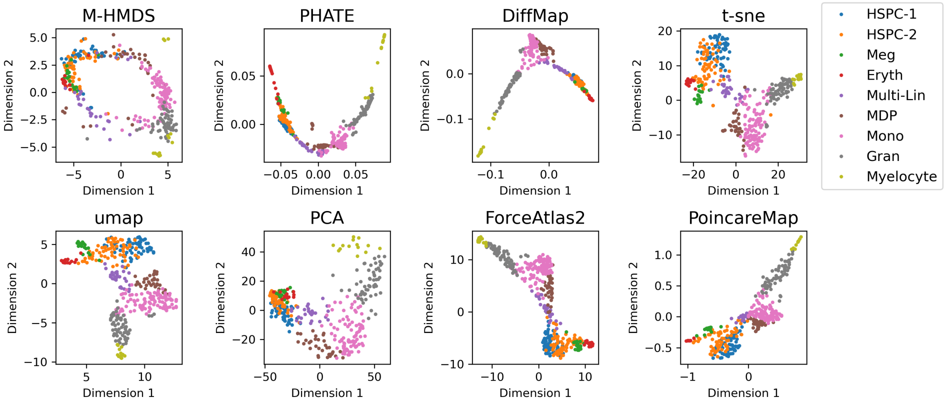

Figure S15: Embedding of the mouse myelopoesis dataset (Olsson), in 2-d space.

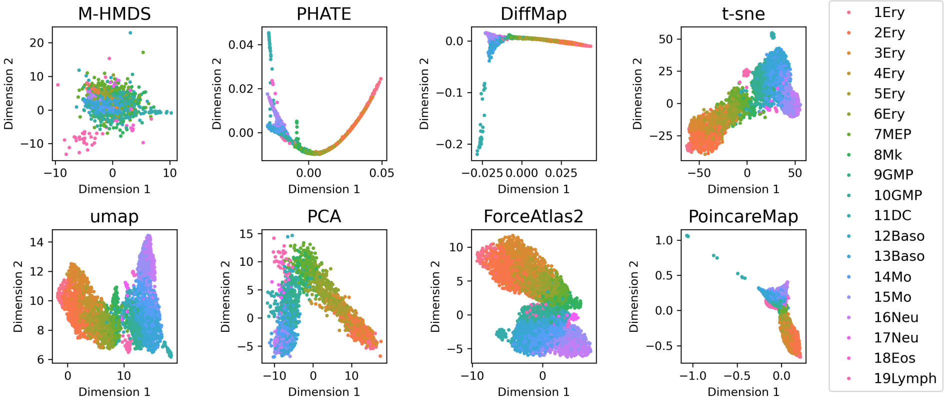

Figure S16: Embedding of the mouse myeloid progenitors dataset (Paul), in 2-d space.

bioRxiv preprint doi: https://doi.org/10.1101/2025.09.29.679407; this version posted October 1, 2025. The copyright holder for this preprint (which was not certified by peer review) is the author/funder, who has granted bioRxiv a license to display the preprint in perpetuity. It is made available under aCC-BY 4.0 International license.

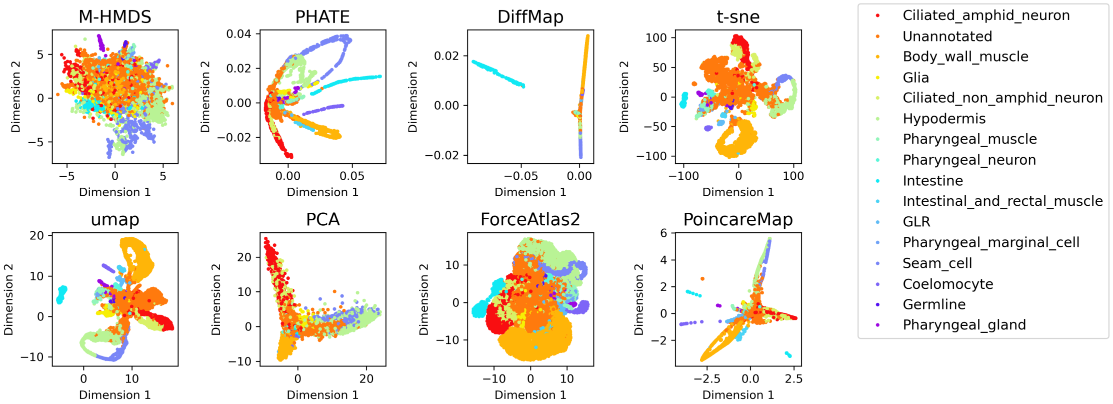

Figure S17: Embedding of the 10,000 sample subset of *C. elegans* dataset, in 2-d space, colored by cell types.

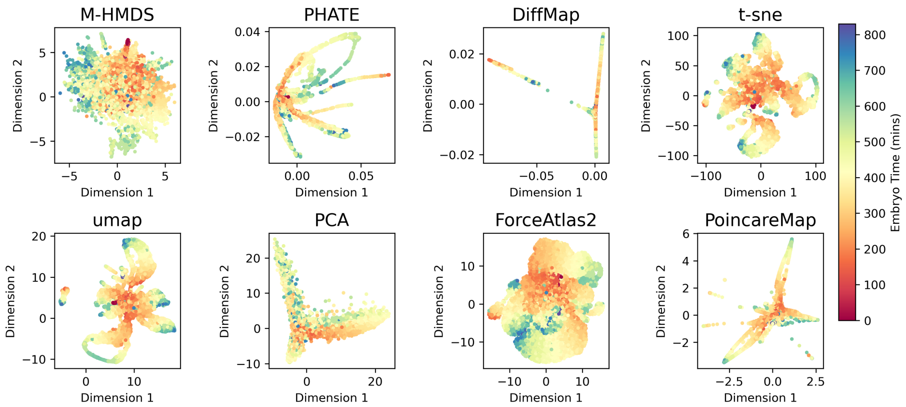

Figure S18: Same as Fig. (S17), but colored by embryo time.

bioRxiv preprint doi: https://doi.org/10.1101/2025.09.29.679407; this version posted October 1, 2025. The copyright holder for this preprint (which was not certified by peer review) is the author/funder, who has granted bioRxiv a license to display the preprint in perpetuity. It is made available under aCC-BY 4.0 International license.

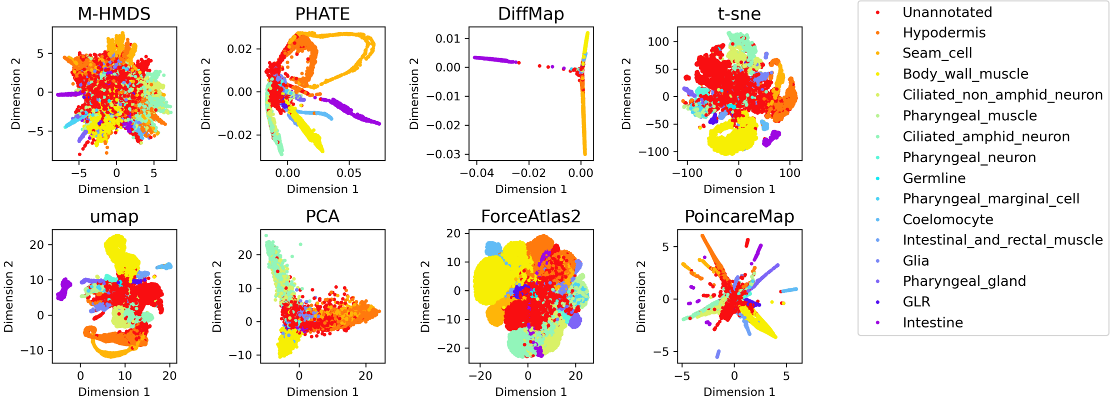

Figure S19: Embedding of the 40,000 sample subset of *C. elegans* dataset, in 2-d space, colored by cell types.

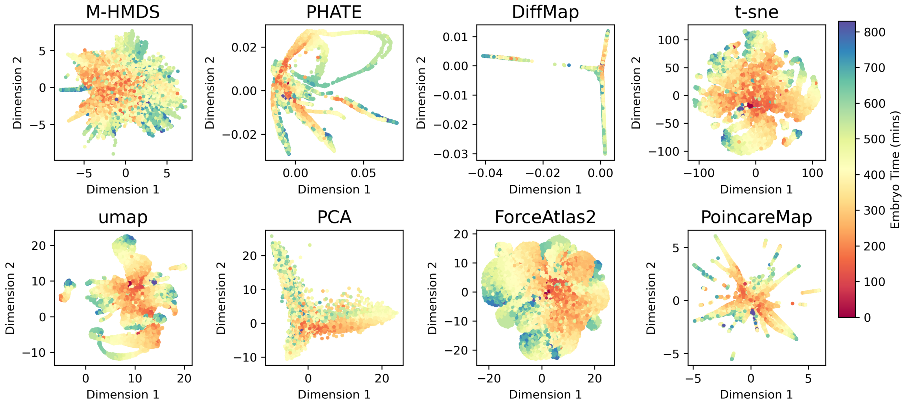

Figure S20: Same as Fig. (S19), but colored by embryo time.
